# Clinical characteristics of patients with atypical teratoid/rhabdoid tumors: a monocentric retrospective analysis

**DOI:** 10.3389/fped.2025.1463510

**Published:** 2025-03-06

**Authors:** Zhiliang Wang, Jingchen Yang, Xing Liu, Wei Liu

**Affiliations:** ^1^ Department of Neurosurgery, Beijing Tiantan Hospital, Capital Medical University, Beijing, China; ^2^ Department of Neuropathology Center, Beijing Neurosurgical Institute, Capital Medical University, Beijing, China

**Keywords:** atypical teratoid/rhabdoid tumor, retrospective study, clinical characteristics, prognostic factors, overall survival

## Abstract

**Purpose:**

Atypical teratoid/rhabdoid tumors (ATRTs) are very rare, highly malignant embryonal neoplasms in central nervous system. The aim of this study was to conduct a retrospective analysis of ATRT patient survival and investigate the prognostic factors associated with ATRT.

**Methods:**

A retrospective study was conducted using information of patients who received treatment between 2016 and 2021 in Beijing Tiantan Hospital. Kaplan–Meier curves were used for overall survival (OS) analysis. Univariate and multivariate COX analyses were applied for OS predicting.

**Results:**

20 histologically confirmed ATRT patients were included. The majority were male (75%) and aged over 3 years (65%). 71.4% of patients under 3 years and 46.2% of above 3 years had supratentorial tumors. All patients underwent surgery, with 60% having total resections, primarily in the supratentorial region. Subsequent treatment involved varying chemotherapy and radiation combinations, with 40% of patients receiving it, and 87.5% of those were older than 3 years, The median overall survival for ATRT patients was 180 days. Survival differed significantly between patients under and above 3 years. Radiotherapy increased overall survival for all patients. Univariate and multivariate analysis showed better survival for those diagnosed above age 3 and with adjuvant radiation.

**Conclusions:**

Patients older than 3 years old had better prognosis and radiotherapy had a significant effect on improving patient prognosis.

## Introduction

Atypical teratoid/rhabdoid tumors (ATRTs) are very rare, highly malignant embryonal neoplasms in central nervous system (CNS) (1). More than 90% cases of ATRT occur in children younger than three years old (2). In addition, ATRT contributes to 40%–50% of malignant CNS tumors in children under one year old, many of which are found disseminated (3, 4). Primitive histologic appearance with multi-lineage differentiation with loss of nuclear SMARCB1 or SMARCA4 expression is a typical histological characteristic of ATRT. Based on the genetic and epigenetic profiles, 3 distinct molecular subgroups-AT/RT-TYR, AT/RT-SHH, and AT/RT-MYC have been recently identified and the prognosis of ATRT seems to differ by 3 molecular subgroups (2, 5, 6).The ATRT-TYR subgroup is enriched in genes associated with melanogenesis and neural crest development, such as TYR and DCT, and is often linked to older children with relatively better prognosis (7). ATRT-SHH is defined by the activation of the Sonic Hedgehog (SHH) signaling pathway, with tumors frequently arising in the cerebellum or midline structures, showing intermediate clinical outcomes (8, 9). The ATRT-MYC subgroup, marked by high MYC expression and other proliferation-related genes, is predominantly found in infants and is associated with the most aggressive behavior and poor prognosis (10, 11). These molecular distinctions have significantly advanced our understanding of ATRT biology and hold potential for subgroup-specific therapeutic strategies.

The current treatment of ATRT is an aggressive multi-modal approach including maximal surgical resection with subsequent adjuvant chemotherapy and radiotherapy (12, 13). In recent years, numerous studies on ATRT were initiated and prolonged the four-year overall survival rate to nearly 50% (14). Specifically, we have noted that megachemotherapy with autoBMT has been employed in protocols such as ACNS0333 (15) and EU-RHAB (16) to improve outcomes in young children with ATRT. Additionally, intraventricular chemotherapy is mentioned as a strategy to address leptomeningeal dissemination, which is common in ATRT (17, 18). These updates provide a more comprehensive overview of current ATRT treatment strategies. However, the pathogenesis of ATRT is biological puzzling and the treatment of it remains a considerable challenge in the world. There are two oncology patient registries at present, including Surveillance, Epidemiology and End Results (SEER) registry and National Center database (NCDB). Studies have reported the epidemiology, prognosis, and survival of ATRT patients by analyzing the data in SEER and NCDB (19, 20). Yet researches based on Chinese patient population are rarely reported.

In this study, we intended to characterize clinical prognostic factors of ATRTs in Chinese patients by retrospectively analyzing ATRT cases collected in Beijing Tiantan Hospital, in the hope of providing novel reference for treatment of ATRT patients.

## The patients and methods

### Patients and cohort

We retrieved 20 patients diagnosed ATRT in Beijing Tiantan Hospital from 2016–2021. The end point of follow-up was January 10th, 2022. Until the end point of follow up (January 10th, 2022), 7 patients were alive. The median time of follow up was 169.5 days. The patients were sorted and analyzed with a series of clinical characteristics including age (under/above 3 years old), gender, tumor location (supratentorial and infratentorial), extent of resection (total/subtotal resection), and subsequential therapy (chemotherapy, radiation, and combination of chemotherapy and radiation).

### Clinical assessment

The diagnosis of ATRT is made by neurosurgeons who combines medical history, imaging features, and pathological examinations. The pathological diagnosis was made by at least 2 experienced neuropathologists. Once the patients with the diagnosis of ATRT was identified, a detailed review of the clinical data was performed to ascertain the presenting features, degree of surgical resection, immunochemistry (IHC) features, and subsequential therapies. The extent of resection was assessed by magnetic resonance imaging (MRI) scanning within 72 h after operation. The IHC features were collected from patients' pathology reports.

Chemotherapy was performed using a 6-cycle of Cisplatin (75 mg/m^2^, 3 days/cycle)—Etoposide (EP, 300 mg/m^2^, 3 days/cycle) regimen alone or during radiotherapy. For patients under 3 years old, focal irradiation was performed regardless of their metastatic status. Patients above 3 years old received 3,600 cGy of craniospinal irradiation (CSI). The dose for focal radiotherapy ranged between 4,500 and 5,500 cGy given for 6 weeks (5 days/week).

### Statistical analyses

Overall survival (OS) was measured from the date of initial diagnosis of ATRT to the date of death or last contact. Kaplan–Meier curves were used for survival analysis and Log-rank test was used to assess the statistical impact of different factors on OS. In comparison with survival estimates in relation to other risk factors, stratification was based on age at diagnosis. Univariate and multivariate COX analyses were applied for OS predicting. *P*-value < 0.05 was considered as significant.

## Results

### Patient characteristics

A total of 20 patients with histologically confirmed ATRT between 2016 and 2021 were identified in our study. The clinical characteristics were shown in Table 1. The majority of the cohort was male (15 patients, 75%) and above 3 years old (13 patients, 65%). Only 1 of the patients was adult (>18 years old). The median age at diganosis was 4 years old and 65% of the patients were older than 3 years. The proportion of supratentorial (11 patients, 55%) and infratentorial (9 patients, 45%) tumors was almost equal. However, supratentorial tumors were more common in patients under 3 years of age (71.4%). For patients above3 years old, there was no significant difference in quantity between supratentorial (46.2%) and infratentorial (53.8%) tumors.

**Table 1:** Integrated characteristics of patients.

Characteristics	Number of patients
Age of diagnosis	
<3	7 (35%)
≥3	13 (65%)
Gender	
Male	15 (75%)
Female	5 (25%)
Tumor location	
Supratentorial	11 (55%)
Infratentorial	9 (45%)
Resection	
Total resection	12 (60%)
Subtotal resection	8 (40%)
Subsequential therapy	
Chemotherapy only	3 (15%)
Radiation only	0
Chemotherapy & Radiation	5 (25%)
No subsequential therapy	12 (60%)

All the patients collected received surgical treatment. 12 (60%) patients underwent total resection, among which 58.3% had the tumors in supratentorial area, while 8(40%) had subtotal resections. Furthermore, the majority of patients received total resection in both under 3 years (71.4%) and above 3 years (53.8%) groups, consisting with the general landscape (21).

Subsequential treatment consisted of different combinations of chemotherapy and radiation. 40% (8 patients) of the cohort received subsequential therapy, all of which received chemotherapy. Meanwhile, 62.5% (5 patients) of the patients who received subsequential treatments underwent radiotherapy. Only 1 patient under 3 years old received subsequential therapy.

### Histological and immunochemical characteristics

Tumors within this patient group displayed a varied range of histopathological and immunohistochemical traits. The study encompassed the examination of Integrase interactor-1 (INI-1), smooth muscle actin (SMA), vimentin (Vim), epithelial membrane antigen (EMA), cytokeratin (CK), and glial fibrillary acidic protein (GFAP). All of the samples analyzed in this investigation exhibited INI-1 deletion in IHC. Moreover, the majority displayed positivity for GFAP, CK, EMA, SMA, and vimentin. Every sample identified exhibited positive signals for SMA. Only 1 (5%) sample demonstrated negativity for Vim. EMA positivity was observed in 84.6% of the detected samples. The percentage of positive results for CK and GFAP stood at 63.2% and 68.4%, respectively (Table 2). These findings were notably consistent with the immune-profiles associated with ATRT (22, 23).

**Table 2:** Immunohistochemical characteristics of ATRTs.

Patient No	INI-1	SMA	Vim	EMA	CK	GFAP
Age of diagnosis <3						
2	-	+	+	NA	+	+
3	-	+	+	-	-	+
4	-	+	NA	+	+	+
10	-	+	NA	NA	NA	NA
11	-	+	NA	+	-	-
15	-	+	NA	+	-	-
20	-	+	NA	+	+	+
Age of diagnosis ≥3						
1	-	+	NA	+	+	+
5	-	+	+	NA	+	+
6	-	+	+	-	+	-
7	-	NA	NA	NA	-	-
8	-	+	+	+	-	+
9	-	+	NA	+	+	+
12	-	NA	NA	NA	+	+
13	-	+	NA	+	-	+
14	-	+	NA	+	+	+
16	-	NA	+	NA	+	-
17	-	NA	+	+	-	-
18	-	NA	-	NA	+	+
19	-	+	+	+	+	+

INI-1, integrase interactor-1; GFAP, glial fibrillary acidic protein; CK, cytokeratin; Vim, vimentin; EMA, epithelial membrane antigen; SMA, smooth muscle actin.

### Survival analysis

**Figure 1:**
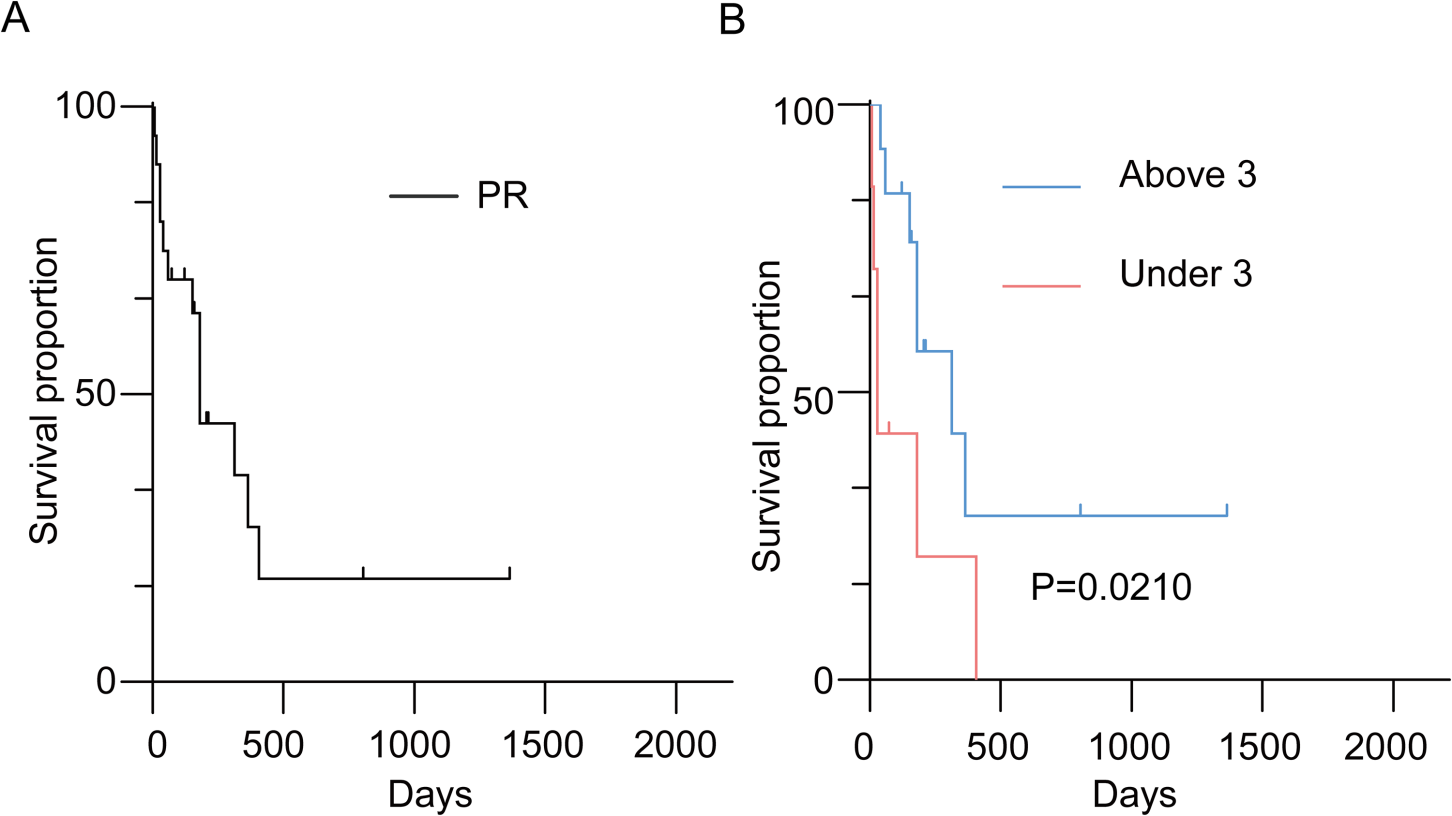
**(A)** Kaplan–Meier survival curve depicting patients with histologically confirmed atypical teratoid/rhabdoid tumors (ATRTs); **(B)** Kaplan–Meier survival curve comparing overall survival in ATRT patients by age dichotomized at three years.

**Figure 2:**
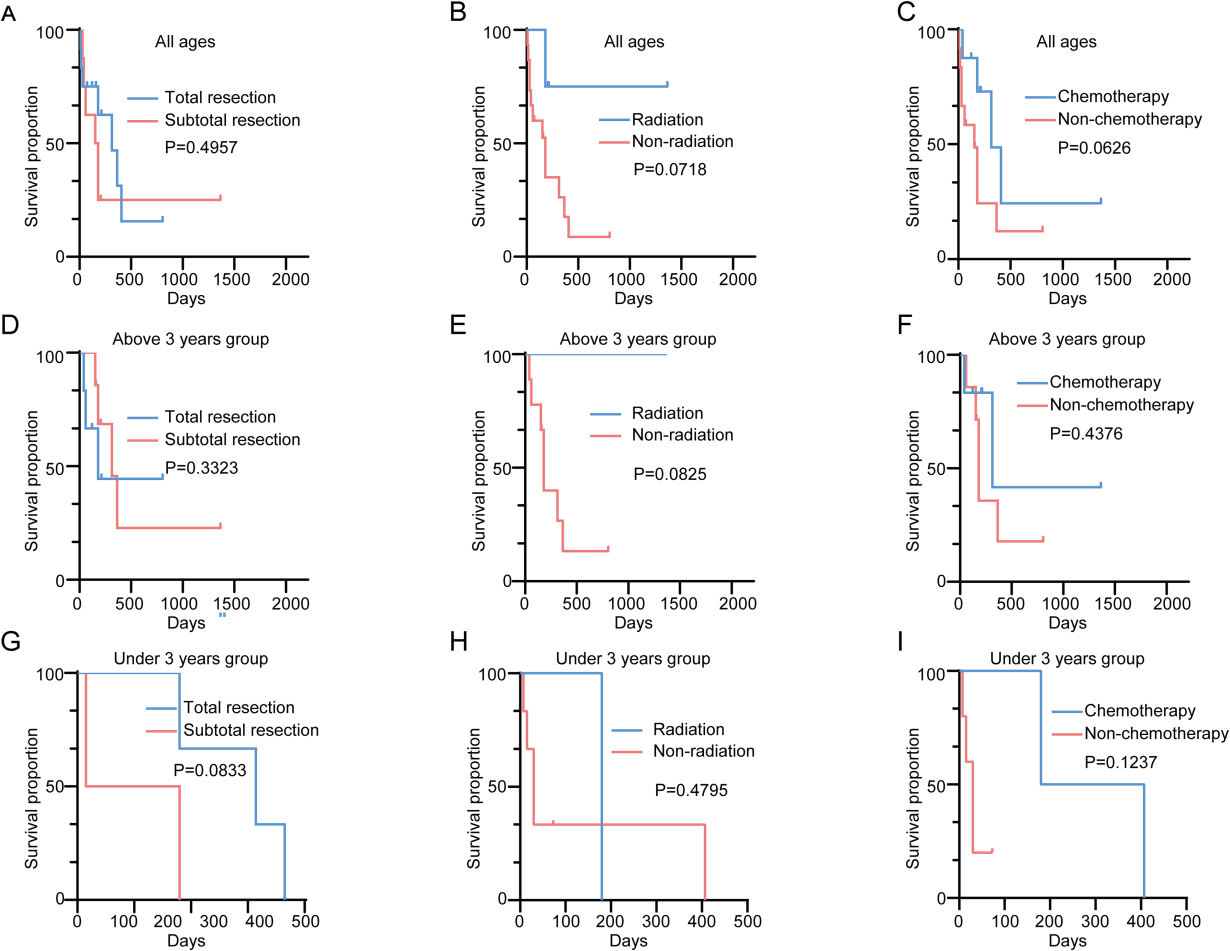
**(A)** Kaplan–Meier survival curve comparing overall survival in ATRT patients receiving total/subtotal resections; **(B)** Kaplan–Meier survival curve comparing overall survival in ATRT patients receiving/not receiving radiation; **(C)** Kaplan–Meier survival curve comparing overall survival in ATRT patients receiving/not receiving chemotherapy; **(D)** Kaplan–Meier survival curve comparing overall survival in ATRT patients above 3 receiving total/subtotal resections; **(E)** Kaplan–Meier survival curve comparing overall survival in ATRT patients above 3 receiving/not receiving radiation; **(F)** Kaplan–Meier survival curve comparing overall survival in ATRT above 3 receiving/not receiving chemotherapy; **(G)** Kaplan–Meier survival curve comparing overall survival in ATRT patients under 3 receiving total/subtotal resections; **(H)** Kaplan–Meier survival curve comparing overall survival in ATRT patients under 3 receiving/not receiving radiation; **(I)** Kaplan–Meier survival curve comparing overall survival in ATRT under 3 receiving/not receiving chemotherapy.

The median overall survival (OS) for ATRT patients was 180 days (Figure 1A). Kaplan–Meier analysis demonstrated a significant difference in median survival between patients under and above 3 years of age (Figure 1B). Notably, the extent of resection did not show significant association with OS overall (Figure 2A). On the contrary, radiation and chemotherapy prolonged the overall survival of ATRT patients (Figures 2B,C). We then segregated the patients into two age groups: above 3 years old and under 3 years old, and conducted a detailed analysis of the correlation between treatment and patients' prognosis. Among patients older than 3 years, radiotherapy continued to enhance overall survival (Figure 2E), whereas the extent of resection and the decision to undergo chemotherapy did not significantly improve patient survival (Figures 2D,F). We were surprised to find that total resection was beneficial for patients under 3 years old (Figure 2G). There was no survival benefit from radiotherapy in patients younger than three years of age (Figure 2H). An extended effect of chemotherapy on survival in patients younger than three years could be observed although no significance was obtained due to the small number of patients (Figures 2G,H).

In univariate analysis, no factor was considered to have a significant effect on prognosis. However, the results of age dichotomization and adjuvant radiation regimen suggested potential survival benefit for those diagnosed with ATRT above the age of 3 and receiving radiotherapy. Subsequential multivariate analysis confirmed the hypothesis that age at diagnosis [HR = 12.241, 95% CI (1.727–86.744), *p* = 0.012] and the administration of radiotherapy [HR = 4.351, 95% CI (0.834–22.708), *p* = 0.031] were associated with an increase in OS (Table 3).

**Table 3:** Univariate and multivariate analysis of OS.

Variables	Univariate analysis		Multivariate analysis	
	HR (95% CI)	*p* value	HR (95% CI)	*p* value
Age at Diagnosis (under/above 3)	0.283 (0.094–0.855)	0.025	0.306 (0.109–0.904)	0.007
Gender	1.882 (0.621–5.704)	0.263	4.967 (0.986–25.008)	0.052
Location	1.025 (0.349–3.005)	0.965	4.263 (0.852–21.345)	0.078
Resection	1.360 (0.471–3.930)	0.570	2.987 (0.604–14.783)	0.180
Radiotherapy	8.096 (1.036–63.258)	0.046	9.265 (1.194–71.892)	0.016
Chemotherapy	2.780 (0.862–8.963)	0.087	2.180 (0.353–13.457)	0.401

## Discussion

ATRT is a rare and aggressive brain cancer, predominantly affecting children. This tumor is recognized for its swift growth and its potential to metastasize to other regions of the central nervous system, rendering it a formidable disease that poses challenges for effective treatment (15, 24).

ATRT primarily impacts young children, typically manifesting in infants and toddlers. Research has consistently shown an elevated risk of tumor development in children under the age of 3, making it a crucial concern in pediatric healthcare (10). The median age of onset of ATRT in the study cohorts reported in European and American countries is mostly less than 2 years old (14, 15). However, the reported diagnosis ages of the ATRT cohort of Chinese patients are relatively older. A study conducted by Shen et al. (25), 10 patients with ATRT were included, 80% of whom were older than 5 years old, and 3 patients were older than 30 years old. In another study conducted by Wang et al. (26), the median age of diagnosis for 15 ATRT patients was 5.5 years, and 53.3% of the patients were older than 3 years. In our study, the median diagnosis age is 4 years old and 65% of the patients were older than 3 years. This is similar to a previously reported patient-based study in China. However, whether the age of Chinese ATRT patients is older than that of European and American patients still need further studies with wider range and larger sample. In addition, while the majority of cases examined in this study involved minors, a significant proportion of patients were over 3 years old. This indicates that precisely determining the age with the highest incidence of ATRT may have some uncertainty. Therefore, during the diagnostic process, especially when dealing with pediatric patients across all age groups, it's essential not to overlook the potential for ATRT. Notably, the survival period for ATRT patients under 3 years old is considerably shorter than for those over 3 years old. Furthermore, this study identified an age below 3 years old as an independent risk factor for ATRT prognosis, consistent with earlier research findings (27–29).

Although there is no uniform guideline, it is widely accepted that the treatment of ATRT typically involves a comprehensive approach, which includes surgical intervention aimed at maximal tumor removal, followed by a combination of radiation therapy and chemotherapy. Achieving gross tumor resection (GTR) through intensive multimodal treatment is a recognized and valuable objective, associated with improved outcomes compared to cases with significant residual disease (7). Our center follows the principle of maximum surgical resection of the tumor. However, in our study, achieving total tumor resection was not identified as an independent factor for enhancing the prognosis of ATRT patients. These further underscores the malignant and highly prone-to-spread and relapse nature of ATRT. It suggests that a simple surgical approach may not effectively enhance patient survival, emphasizing the need for a multidisciplinary and comprehensive treatment approach.

Currently, the exploration of comprehensive treatment strategies for ATRT, combining surgery with chemoradiotherapy, is actively ongoing. This includes research on treatment guided by molecular typing of ATRT, which has garnered considerable attention (10, 11). The future of ATRT treatment lies in multidisciplinary and comprehensive approaches. In our study, the patients were treated with 6-cycle of Cisplatin-EP regimen alone or during radiotherapy at the center. In addition, the center stratified patients according to thier age, and developed different radiotherapy programs for patients of different age groups. We observed that overall, patients who underwent radiation therapy exhibited significantly higher survival rates compared to those who did not. Similar results were seen in patients over 3 years old, although this wasn't the case for those under 3 years old. This discrepancy may be attributed to the limited number of patients below 3 years of age receiving radiation therapy. Notably, while chemotherapy did not independently improve outcomes for ATRT patients overall, it notably extended survival for patients under 3 years old. Importantly, all patients who received radiotherapy also received chemotherapy in this study. Although not covered in this study, high dose chemotherapy and stem cell transplant are also important treatments for ATRT patients and are becoming more widely recommended and used, especially for young children (30–32). Several studies have reported promising effects of multiple treatments, including high dose chemotherapy and auto stem cell transplantation, in prolonging survival in patients with ATRT. A study by Yamada et al. put forward that ATRT patients with localized tumors without germline *SMARCB1* aberrations can be rescued with multimodal therapy, including induction therapy containing ICE followed by HDCT/autoPBSCT and intrathecal topotecan maintenance therapy without radiotherapy (33). Griffith-Linsley et al. reported one case of ATRT with prolonged survival after auto stem cell transplantation treatment, and reviewed the literature on the benefit of auto stem cell transplantation treatment in four adult ATRT patients (31). Although larger studies are still needed, high dose chemotherapy and auto stem cell transplantation have promising prospects in the treatment of ATRT. Hence, treating ATRT necessitates a comprehensive approach involving multidisciplinary collaboration and a range of professionals, building upon the foundation of surgery (34).

Limitations of this study lie in its retrospective characteristics and insufficient number of cases and further prospective studies are required to validate conclusions. In addition, as an important factor affecting patient survival (34), stage statistics was not included in this study. Furthermore, this study did not collect molecular typing data of patients. Genetic testing could provide strong support for tumor diagnosis and classification, and additional insights into the underlying molecular mechanisms (35). Recent research demonstrated that ATRT subgroups are associated with distinct genotypic, chromatin, and functional landscapes that correlate with cellular responses to various signaling and epigenetic pathway inhibitors; compounds specifically targeting these pathways or agents that alter the epigenetic state of the cell are currently being evaluated (10, 11, 36). Future studies may further investigate anatomical, functional, and genetic characteristics of molecular subgroups during treatment and surveillance of ATRT in a prospective fashion.

## Data Availability

The raw data supporting the conclusions of this article will be made available by the authors, without undue reservation.
